# Development of Machine Learning Models for Accurately Predicting and Ranking the Activity of Lead Molecules to Inhibit PRC2 Dependent Cancer

**DOI:** 10.3390/ph14070699

**Published:** 2021-07-20

**Authors:** Danishuddin, Vikas Kumar, Shraddha Parate, Ashutosh Bahuguna, Gihwan Lee, Myeong Ok Kim, Keun Woo Lee

**Affiliations:** 1 Department of Bio & Medical Big Data (BK21 Program), Division of Life Sciences, Research Institute of Natural Science (RINS), Gyeongsang National University (GNU), 501 Jinju-daero, Jinju 5282, Korea; danish.info16@gmail.com (D.); vikaspathania777@gmail.com (V.K.); 2 Plant Molecular Biology and Biotechnology Research Center (PMBBRC), Division of Applied Life Science, Gyeongsang National University (GNU), 501 Jinju-daero, Jinju 52828, Korea; parateshraddha@gmail.com; 3 Department of Food Science and Technology, Yeungnam University, Gyeongsan 38541, Gyeongsangbuk-do, Korea; ashubahuguna@gmail.com; 4 Division of Applied Life Sciences, Gyeongsang National University (GNU), 501 Jinju-daero, Jinju 5282, Korea; pika0131@naver.com; 5 Division of Life Science and Applied Life Science (BK 21 Four), College of Natural Sciences, Gyeongsang National University, Jinju 5282, Korea

**Keywords:** cancer, epigenetic, PRC2, machine learning, multi-class models

## Abstract

Disruption of epigenetic processes to eradicate tumor cells is among the most promising interventions for cancer control. EZH2 (Enhancer of zeste homolog 2), a catalytic component of polycomb repressive complex 2 (PRC2), methylates lysine 27 of histone H3 to promote transcriptional silencing and is an important drug target for controlling cancer via epigenetic processes. In the present study, we have developed various predictive models for modeling the inhibitory activity of EZH2. Binary and multiclass models were built using SVM, random forest and XGBoost methods. Rigorous validation approaches including predictiveness curve, Y-randomization and applicability domain (AD) were employed for evaluation of the developed models. Eighteen descriptors selected from Boruta methods have been used for modeling. For binary classification, random forest and XGBoost achieved an accuracy of 0.80 and 0.82, respectively, on external test set. Contrastingly, for multiclass models, random forest and XGBoost achieved an accuracy of 0.73 and 0.75, respectively. 500 Y-randomization runs demonstrate that the models were robust and the correlations were not by chance. Evaluation metrics from predictiveness curve show that the selected eighteen descriptors predict active compounds with total gain (TG) of 0.79 and 0.59 for XGBoost and random forest, respectively. Validated models were further used for virtual screening and molecular docking in search of potential hits. A total of 221 compounds were commonly predicted as active with above the set probability threshold and also under the AD of training set. Molecular docking revealed that three compounds have reasonable binding energy and favorable interactions with critical residues in the active site of EZH2. In conclusion, we highlighted the potential of rigorously validated models for accurately predicting and ranking the activities of lead molecules against cancer epigenetic targets. The models presented in this study represent the platform for development of EZH2 inhibitors.

## 1. Introduction

Epigenetic mechanisms are crucial for normal development and maintenance of tissue-specific gene expression [1]. Disruption of epigenetic processes can lead to altered gene function and malignant cellular transformation [1,2]. Polycomb repressive complex 2 (PRC2) is a histone methyltransferase complex composed of core subunits EZH2, EED, Suz12 and Rbbp4, forming a stable and enzymatically active methyltransferase complex [2,3,4,5,6]. Enhancer of zeste homolog 2 (EZH2) is the catalytic component of polycomb repressive complex 2 (PRC2), that exhibits an intrinsic trimethylation activity on lysine 27 of histone H3 (H3K27) [6,7,8]. EZH2 overexpression is frequently found in various human cancers including breast, prostate, and bladder cancer [5,6]. Mutation at Y641 was found to increase activity of PRC2 on H3K27me2 substrates and greatly reduce activity on H3K27me0 substrates [9,10]. In contrast, mutation at A677G results in an almost equal preference of PRC2 for all the methylation states [9]. Different types of EZH2 inhibitors have been developed; most of them are under evaluation in clinical trials [11,12,13,14]. Pharmaceutical companies including GlaxoSmithKline (GSK), Novartis, Epizyme and research institutions have done extensive work and revealed a competitive small molecule, which potently suppresses function of EZH2 [15,16,17,18,19]. Compounds GSK126 and EPZ005687 are highly selective and potently inhibit wild-type and lymphoma-associated mutants of EZH2 [17,18,19]. These compounds share very similar pharmacophoric features and are fairly selective for EZH2 versus EZH1. In addition, GSK343 also inhibits EZH2 with similar potency [20]. El1 was optimized from a hit of a high-throughput screening at Novartis and has structural features and selectivity similar to that of EPZ005687 and GSK126 [21]. However, this compound did not have any in vivo activity. Compound UNC1999 is similar in structure to GSK343, known to be first orally bioavailable inhibitor of EZH2, and is the most panactive EZH2 inhibitor to date [22]. Biochemical analysis shows that these inhibitors block EZH2 catalytic activity through a cofactor S-adenosylmethionine (SAM)-competitive mechanism rather than disruption of PRC2 complex formation. Although these inhibitors show some potential outcomes, one of the major limitations, however, is the structure diversity among them, as they have a similar SAM-like scaffold. Moreover, it has also been highlighted that some of the EZH2 mutations easily lead to resistance against these drugs [23,24]. Recently, efforts have been made to discover EZH2 inhibitors with novel scaffold [25,26,27,28]. Thus, available information can provide an advantage in developing the classification or predictive models. We also define the chemical space in the search for versatile inhibitors against EZH2, guiding the fast and reliable generation of novel scaffold agents able to act through different mechanism of action.

In this study, we have developed prediction models for EZH2 by applying a variety of machine learning and feature selection techniques. The well validated models were used to screen the chemical libraries to identify the novel potential hits with broad spectrum active against EZH2. Thus, we identified potent EZH2 inhibitors with novel scaffolds using ligand and structure-based docking approaches. Results from this study can also guide further development of novel specific EZH2 inhibitors.

## 2. Results

### 2.1. Model Development and Evaluation

**Figure 1:**
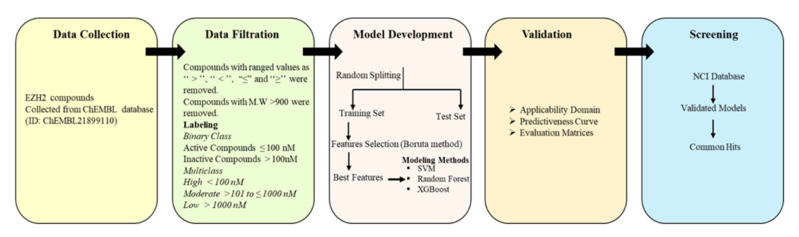
A framework guiding the development and evaluation of a predictive model for EZH2. The stages are: (1) Data collection, (2) Data cleaning and threshold setting, (3) Development of models, (4) Validation of the model and analysis, (5) Screening of chemical libraries using the validated models.

**Figure 2:**
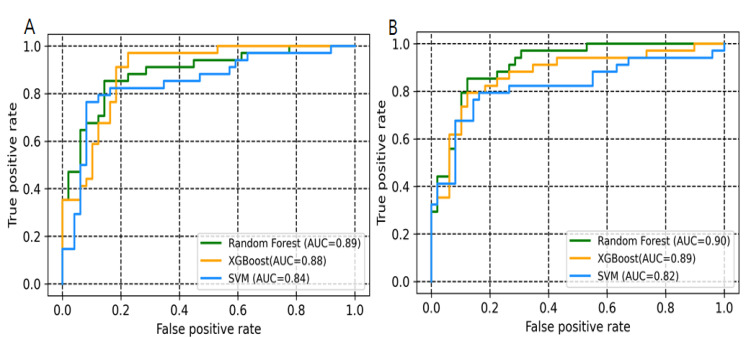
ROC curve showing the performance of binary class models. (**A**) All descriptor set. (**B**) Selected descriptors set (Boruta method).

**Figure 3:**
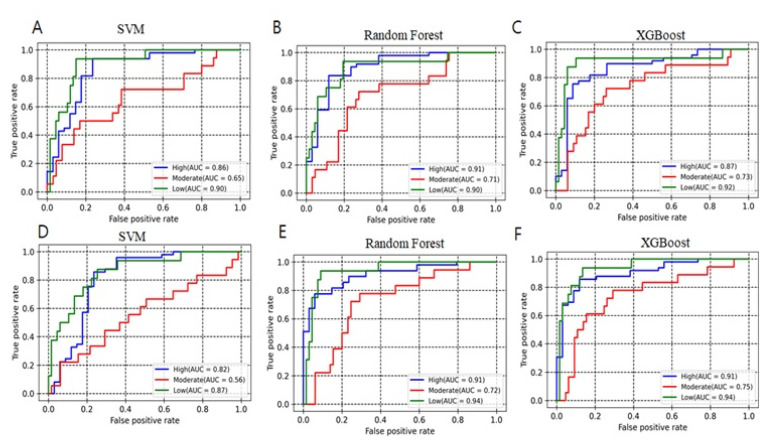
ROC curve showing the performance of multiclass models. (**A**–**C**) All descriptors set. (**D**–**F**) Selected descriptors (Boruta method).

**Figure 4:**
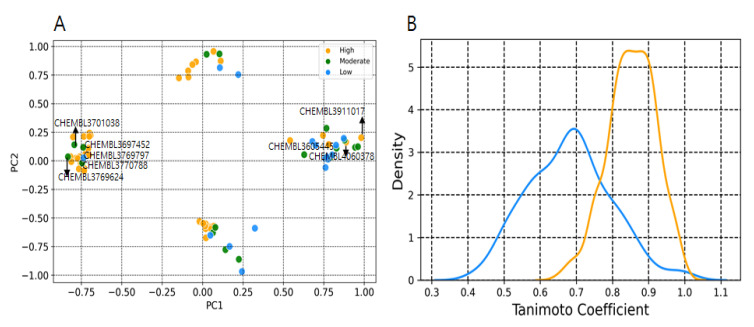
Plot showing the chemical space of falsely predicted compounds and their Tc similarities. (**A**) Distribution of falsely predicted compounds from test set. (**B**) Density plots of Tc values using MACCS fingerprints. Compared were falsely predicted low and moderate vs. high activity compounds (Orange color), and falsely predicted high activity compounds vs. low and moderate activity compounds of training set (Blue color).

A workflow for the EZH2 modelling process is shown in Figure 1. The binary and multiclass models were trained using three machine learning methods (SVM, random forest and XGBOOST) along with all descriptors set (descriptors selected after using the variance and correlation filters) and descriptors selected from Boruta’s method [29]. The descriptors selected from Boruta method were listed in Table 1. The following quantities were used for model building: Total, ~412; Active: 217; Inactive: 195 (Moderate, 88; and Low activity, 107). Performance of developed models is given in Table 2 and Table 3, including the accuracy, recall, precision, F1 and AUC values. Random forest and XGBoost performed with a better statistic in combination with the selected descriptor set. For binary class models, random forest and XGBoost with all descriptors set achieved an accuracy of 0.79 and 0.78, respectively. Improvement has been noticed with selected Boruta descriptors set where random forest and XGBoost achieved an accuracy of 0.80 and 0.82, respectively (Table 2). For multiclass classification, the performance of random forest in accuracy, macro-averaging precision, macro-averaging recall, macro-averaging F1 score is 0.73, 0.63, 0.62 and 0.62, respectively; for XGBoost, the performance was 0.75, 0.67, 0.67 and 0.67, respectively. Random forest and XGBoost models had a significant AUC value for both binary and multiclass class models (Figure 2 and Figure 3). The AUC values for moderate class were lower as compared to high and low activity classes (Table 3 and Figure 3). SVM was found to decrease the prediction performance in combination with selected descriptors set. Ten compounds were commonly predicted falsely by random forest and XGBoost methods. Figure 4A shows the distribution of falsely predicted compounds on principle components analyses: PC1 and PC2 coordinates (based on eighteen descriptors set). These compounds share reasonable similarities with either class (Figure 4B). The average Tanimoto coefficient (Tc) similarity value of these falsely predicted compounds with the high activity compounds in training set was found to be 0.81, whereas the average Tc similarity value of falsely predicted low and moderate activity compounds was 0.68 (Figure 4B). Low activity compound (CHEMBL3769791) which was predicted as active by both the methods has an average Tc similarity value of 0.80.

### 2.2. Y-Randomization and Applicability Domain

**Figure 5:**
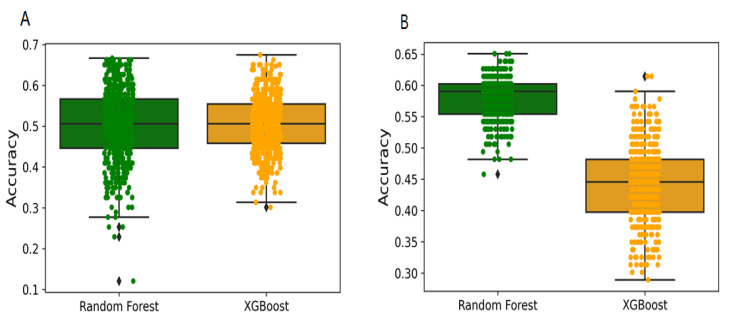
Box plot showing the frequencies of accuracies from Y-randomization models. (**A**) Binary class models. (**B**) Multi-class models. Total 500 Y-randomization runs were performed.

**Figure 6:**
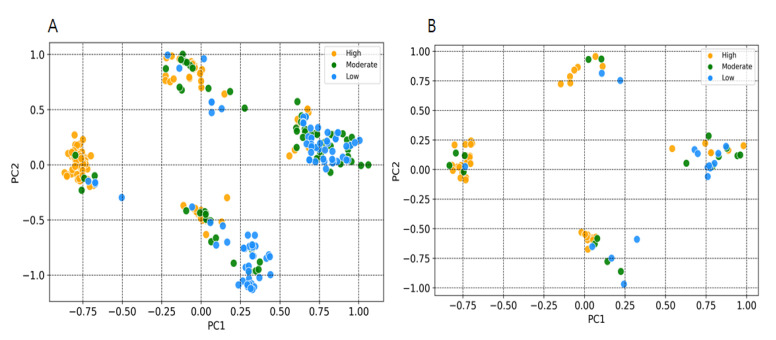
Applicability domain plot based on principal component analysis (PCA) using eighteen selected descriptors set. (**A**) Training set. (**B**) Test set.

Y-randomization was used for assessing the risk of obtaining classification models by chance correlation [30]. In our case, accuracies for Y-randomization test were found to be lower, and none of the 500 random trials achieved a performance higher than our original models (Figure 5A,B). The average accuracies of all random models were found to be less than 0.58, which confirm that the selected predictive models are robust and reliable and not generated with by-chance correlations. An applicability domain (AD) experiment was performed to check the reliability of developed models. Figure 6A,B depict the scatter plot of the PC1 and PC2 coordinates derived from selected descriptors set. The result from this approach shows that the training and test compounds (except few) share similar PC1 and PC2 coordinates, suggesting that predictions were in the applicability domain (AD) of both the training and test sets. A pairwise comparison of the compounds in each cluster reflects reasonable Tanimoto coefficient similarities between them.

### 2.3. Probabilistic Distribution

**Figure 7:**
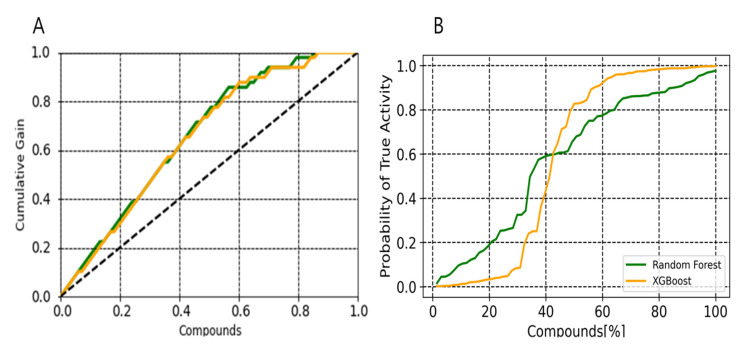
Probabilistic distribution plots. (**A**) Cumulative gain plot for random forest (green line) and XGBoost (orange line) models (**B**) Predictiveness curves. Both the plots were plotted using the selected descriptor set.

Cumulative gain plot was used to visually assess the performance in early recognition of hits of a predictive model [31,32]. Comparison shows that both the methods perform similarly in terms of early recognition of hits (Figure 7A). We have used a predictiveness curve to define a probability threshold for which we can compute that a molecule with this given probability score will be active hits (Figure 7B). Differences in activity probabilities allowed us to quantify the predictive or discriminating power of each classifier. The contributions of a selected descriptor set for predicting the active compounds was quantified using the total gain (TG) and partial total gain (pTG). Total gain (TG) for XGBoost and random forest was found to be 0.79 and 0.596, respectively. The partial total gain of 0.488 in the selected subset illustrates that each compound in this subset has an average probability gain of 0.488 of being active over the random picking of compounds. The prevalence for XGBoost was 0.578 and for random forest the prevalence was 0.602, whereas Brier score was 0.180. The Brier score is a well-defined metric used to measure the mean squared distance between the observed and predicted outcomes on the probability scale. The Brier score is more akin to a cost function where the lower the values, the better the predictions are. The value of the Brier score confirmed that these selected models are well calibrated, thus robust for further predicting active and potent EZH2 compounds from an external database reasonably well.

### 2.4. Virtual Screening and Molecular Docking

**Figure 8:**
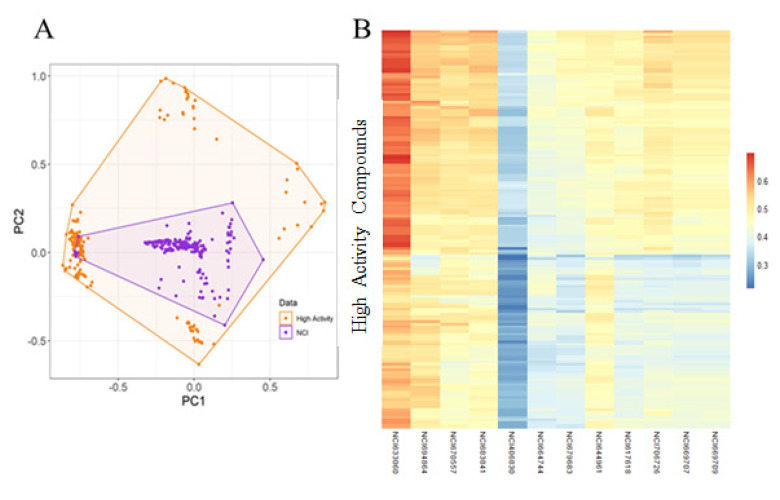
Library screening result. (**A**) Chemical space of selected active hits. (**B**) Heatmap showing the Tanimoto similarities score (Tc) of selected compounds with high activity compounds from training set. MACCS fingerprints were used to calculate the Tanimoto coefficient similarities.

**Figure 9:**
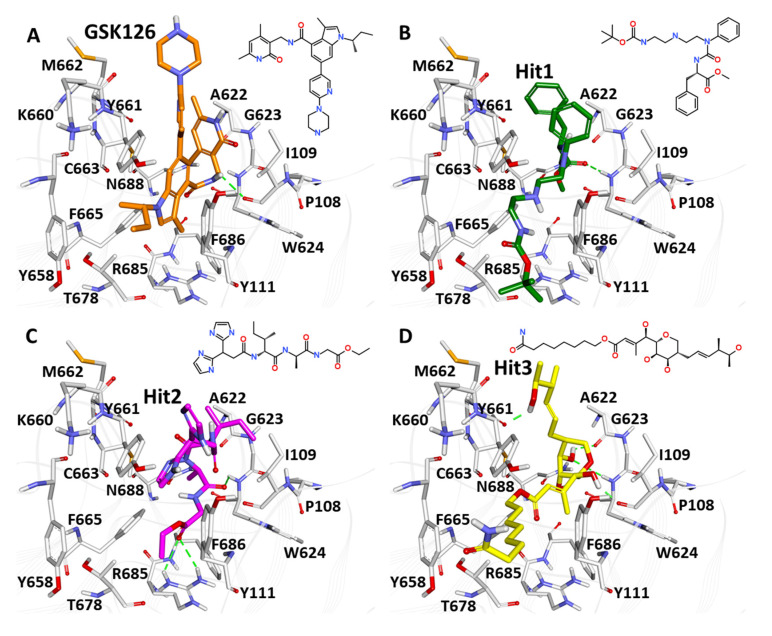
Binding analysis of top hits from molecular docking: (**A**) Reference compound (GSK126), (**B**) Hit1 (NCI694864), (**C**) Hit2 (NCI670557) and (**D**) Hit3 (NCI706726). The active site residues are shown in grey sticks. The protein backbone is shown in light grey wire. Hydrogen bonds were illustrated with green dashed line.

**Figure 10:**
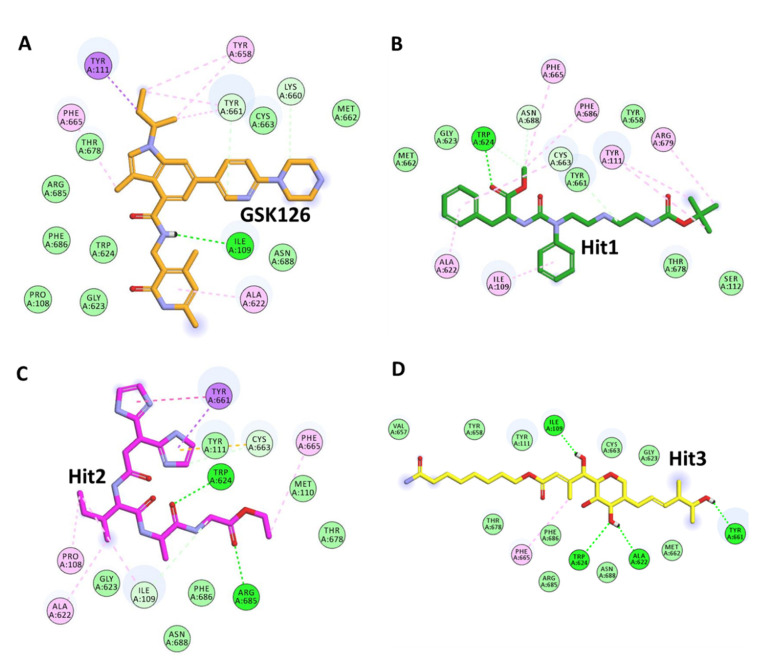
Two-dimensional schematic representations of protein–ligand interactions between GSK126 (reference compound) and the top three hits: (**A**) GSK126, (**B**) Hit1 (NCI694864), (**C**) Hit2 (NCI670557) and (**D**) Hit3(NCI706726). The hydrogen bonds, Л-Л, Л-cation, and Л-Sulphur are shown as green, pink and purple dashed lines, respectively.

NC1 (National Cancer Institute) has been used for screening the hits from validated models. Commonly predicted active compounds with set probability score from validated models were selected and further filtered out with applicability domain (AD) of high activity compounds from the training set (Figure 8A,B). A total of ~221 compounds were found to be under the applicability domain (AD) and were further subjected to docking simulation. Three compounds (NCI694864, NCI670557 and NCI706726) were observed to have reasonable binding affinity and stable interaction with the catalytic residues in the active site (Table 4, Figure 9 and Figure 10). The compound NCI706726 was found to stabilize the complex through four hydrogen bonds with Ile109, Ala622, Trp624 and Tyr661; compounds, NCI670557 and NCI706726 were found to stabilize the complex with two hydrogen bonds each. These compounds have a similar binding mode with GSK21. The Tanimoto coefficient (Tc) similarity score of these selected hits was found to be ≤0.5 with high activity compounds (Figure 8B). 

## 3. Discussion

EZH2 is frequently overexpressed in several cancers, promoting cancer cell proliferation and survival. Considering the importance in cancer therapeutics, researchers from both academic and industries have focused on exploring the structural and biological function of PRC2. During the past decade a number of groups have actively continued to develop potential lead molecules for EZH2. Most of these inhibitors are known to inhibit the EZH2 enzymatic activity through a cofactor S-adenosylmethionine (SAM)-competitive mechanism rather than disruption of PRC2 complex formation. In addition, some groups have focused on designing inhibitors that can target the EED-EZH2 interface, which leads to PRC2 inactivation [27,33,34]. This study aimed to build an appropriate classification model for predicting potential hits for EZH2. We have built two types of classification models, including binary and multiclass with three different machine learning approaches. Our proposed models based on random forest and XGBoost performed well in terms of accuracy, F1 score, precision, and recall. For comparisons of classifiers, we used the area under the receiver operating characteristic curve method. A ROC curve is a graphical plot that illustrates the true positive rate against the false positive rate of classifier at different threshold settings. The AUC is then the area under this curve which represents the degree of separability and significant metrics for evaluating machine learning algorithms [35]. Higher AUC values in the ROC curves infer greater sensitivity of retrieving high activity compounds and specificity for ignoring low and moderate activity compounds (Figure 3). Based on feature importance, our study also distinguished and ranked the top eighteen variables including 2D autocorrelation, Burden modified eigenvalues, topological charge, MACCSFP105 and MACCSFP114, etc. These descriptors have capabilities to distinguish between high and low activity compounds. As shown in Figure 3, these descriptors separated the high activity and moderate or low activity compounds into different clusters. Only one low activity compound (CHEMBL3769791) was found to be in a cluster having a large number of high activity compounds. Similarly, only four high activity compounds were found to cluster with low or and moderate activity compounds (Figure 5A). The importance of these type of descriptors has also been highlighted in previous studies which focused on modelling the anti-cancer compound activity [36]. Falsely predicted compounds were found to have reasonable similarities with the true class (Figure 4B). Taken together, these results demonstrate the importance of these descriptors’ subset for predicting the activity of EZH2 compounds at lower range. 

The proper inclusion threshold values of IC_50_ values can improve the prediction. Therefore, it may be desirable for the dataset to have an appropriate activity threshold in the construction of predictive models. The threshold at which molecules are labelled determines the fraction of data points belonging to the “active” or “inactive” class. Generally, 10 μM is a commonly used threshold for distinguishing activity vs. inactivity in any classification model development procedure [37,38,39]. However, this can lead to prediction of a high fraction of active compounds, which is not in accordance with what is observed experimentally [40,41]. Moreover, in an experimental context, model output should ideally lead to identification of compounds with affinity higher than 10 μM to make the most efficient use of costly experimental validation. Thus, to make sure that our predictive models are as reliable as possible, we chose to limit the threshold to a minimum, including for active compounds IC_50_ ≤ 0.1 µM, inactive compounds >0.1 µM. Setting such thresholds can be helpful in identifying the high affinity molecules and in predicting the inhibitory effects of inhibitors based on molecular descriptors [42]. 

Y-randomization was used to evaluate the risk of a random correlation in a selected descriptor. This method is known to be one of the most powerful validation procedures for QSAR models and is used to evaluate the reliability or robustness [43,44]. We have performed 500 randomization runs to evaluate the reliability of our developed models. The statistical significance of a developed model was checked by comparing its performance to the average measure of random models that are obtained using randomly scrambled target variable class and applying the same parameters as those used in building the original model. Y-randomization test confirms that the models developed in this study did not display a correlation by chance, and that there is a true structure–activity relationship (Figure 5A,B). According to the OECD guiding principles, a QSAR model should have a defined domain of applicability [45]. In order to obtain models with a wide applicability domain (AD), the dataset should be chemically diverse and have wide chemical space. We have used a principal component analyses-based approach [46] to define the AD of EZH2 compounds used for modelling purposes. The 2D plots from first two principal components (PC) highlight that training and test sets compound cover similar chemical space and structural diversity, which provides enough confidence in the models being developed (Figure 6A,B).

The predictiveness curve is a metric usually used in clinical epidemiology to evaluate the ability of a biological marker to assess the fit of risk models and to estimate the clinical utility of a model when applied to a population [47,48,49]. This metric can be used to assess the predictive power of a classifier as well as defining a probability threshold, the retrieving best candidates to be tested experimentally. In our study, we preferred to rationally select an optimal probability threshold rather than selecting an arbitrary fraction of the top scoring compounds. We have used PC to define a probability scoring threshold for which we can compute the probability that a molecule with this given score will be active hits. In an ideal case where all active compounds had better probability than the inactive compounds, the threshold would simply be defined as the value separating the probability values of high ranked active compound and the low ranked compound. We found that XGBoost in comparison with random forest performed better in high probability of active compounds (Figure 7). We believe that PC metrics can take into account the probability scores calculated from classifier to better understand results, which may also support the enhancement of the performances of any predictive models. 

Three compounds (NCI694864, NCI670557 and NCI706726) were found to have reasonable binding affinity with EZH2 as compared to known inhibitor (Table 4). They make stable complexes interacting with crucial catalytic amino acids through the hydrogen bonds and hydrophobic interactions. Compound NCI694864 forms two hydrogen bonds with Trp624 and Cys663 residues. The backbone of Trp624 is derived from the conserved GXG motif of the SET domain and is crucial for ligand binding through hydrogen bonds [50]. Another important residue, Tyr111, was found to interact with these hits through hydrophobic interaction. Similar interactions were also reported in previously published studies which highlighted the importance of these amino acids in PRC2 complex formation and inhibitor recognition [5]. Therefore, the three selected compounds comprising aforementioned scaffolds can be considered as a novel source for future identification of PRC2-EZH2 complex inhibitors with novel mechanisms of action and different chemical features.

## 4. Materials and Methods

A workflow for the EZH2 modelling process was shown in Figure 1. The EZH2 activity data were collected from the ChEMBL database [51]. Compounds with ranged values such as ‘‘>’’, ‘‘<’’, ‘‘≤’’, and ‘‘≥’’ were removed from the dataset. If a single compound had more than one IC_50_ value, then the highest of their values was taken into consideration. Compounds with IC_50_ ≤ 0.1 μM were set as active, whereas compounds with IC_50_ values > 0.1 μM were set as inactive molecules. In addition to binary class, we also performed multiclass modelling and compounds with IC_50_ ≤ 0.1μM were labelled as “High”, IC_50_ > 0.1 μM to IC_50_ ≤ 1 μM as “Moderate”, and IC_50_ >1 μM as “Low” affinity. Compounds with >1000 M.W were also removed. A total of ~412 compounds (High activity: 217, Moderate: 88, and Low activity: 107) were selected for predictive model building. Finally, the remaining compounds were randomly divided into training sets and test sets by an 80:20 ratio. 

In total, 83 compounds have been used for validation (Active: 49, and Inactive: 34).

### 4.1. Descriptor Calculation and Selection

Molecular descriptors and fingerprints have been routinely used for quantitatively or qualitatively representing the structural features of a drug [52,53]. Molecular descriptors and fingerprints were calculated using the PaDEL software [54]. To avoid the overfitting chance the number of descriptors was reduced with the following criteria: (1) Descriptors with small variance (<0.2) were removed. (2) For any pair of descriptors with a >0.85 correlation, one descriptor was removed randomly. (3) Finally, the Boruta algorithm [29] was used to selected the best subset of descriptors for model building. 

### 4.2. Model Building

Model building was performed with Python TensorFlow and Kera’s platform [55]. Three machine learning techniques including support-vector machine (SVM) [56], random forest [57] and XGBoost [58] were applied for model building. For SVM, the radial basis function kernel was used, and penalty parameter C was set to 8.0. In the random forest, five hundred trees were used, and a default mtry value was applied. One-versus-All (OvA) was used for multiclass modelling. To accommodate for imbalanced datasets, Scikit class weight scheme was set to balance the classes. Different weights have been assigned to both the majority and minority classes. The purpose of doing this step was to penalize the misclassification made by the minority class by setting a higher-class weight and at the same time reducing weight for the majority class. A 5-fold cross-validation (5-fold CV) was performed with the sub-training set to identify the best model estimators. Finally, the external predictivity of the models was estimated with the test set.

### 4.3. Model Validation

The performance of models on the independent test set was evaluated with a 5-fold cross-validation. The entire dataset was first divided into k non-overlapping subsets, where the first subset was used as a validation set for a model trained on the remaining k-1 subsets. This procedure was repeated k times, employing different subsets as the validation set. Averaging the performance obtained for all k subsets yields the overall performance with the estimated validation error of the model. Precision-macro, recall-macro and F1-macro metrices were used for multiclass models’ evaluation. Such matrices are more sensitive toward the class imbalance and recommended for multiclass models’ evaluation [59]. Model quality was also assessed with the receiver operating characteristic (ROC) plot and area under the curve (AUC) [35]:

(1) $$Precision_{Macro}=\frac{\sum_{i}^{n}\frac{TP_{i}}{TP_{i}+FP_{i}}}{n}$$

(2) $$Recall_{Macro}=\frac{\sum_{i}^{n}\frac{TP_{i}}{TP_{i}+FN_{i}}}{n}$$

(3) $$F1_{Macro}=\frac{2*Precision_{Macro}*Recall_{Macro}}{Precision_{Macro}+Recall_{Macro}}$$

(4) $$BrierScore=\frac{1}{N}\overset{n}{\underset{1}{\sum }}{\left(P_{p}-O_{p}\right)}^{2}$$

where, *P_p_* is predicted probabilities and *O_p_* is observed probabilities.

### 4.4. Predictiveness Curve

Predictiveness curve was used to assess the performance of developed models and confidence of the predicted compounds [60]. We used total gain (TG) and partial total gain (pTG) to quantify the predictive power of models. Summary measures of the predictiveness curve address the need to compare one or more tests statistically, or to concisely summarize the predictive performance of the models.

### 4.5. Applicability Domain

A well validated predictive model requires a defined applicability domain (AD) for highlighting a part of the chemical space containing those compounds for which the model is supposed to provide reliable predictions [61]. Any predictive model needs to confirm the limitations with respect to its structural domain and response space. Therefore, the problem of defining the AD of a model is closely related to the problem of assessing the reliability of its predictions. Generally, such QSAR models cover defined chemical space on the basis of the training set. If any query compound falls within this define AD, prediction of the model is reliable. Otherwise, the prediction may not meet the model’s assumptions. In our study, we have used principal component analyses (PCA) to define the AD of the selected descriptors set [62].

### 4.6. Y-Randomization

Y-randomization was used to check the robustness of the developed models. This method consists of randomly shuffling the values of the target variable in the training set [30,44]. Then, a new prediction was developed using the scrambled data with the same parameters as used in the original modelling. Every run estimates the accuracy of the model were recorded. We performed the 500 runs Y-randomization test using 50% of the target class. 

### 4.7. Similarity Calculations

MACCS fingerprints were used to calculate the systematic pairwise similarity of the selected compounds. All statistical analysis was performed using R Statistical Software (version 4.0.4) and RDKit framework [63].

### 4.8. Library Screening and Molecular Docking

The selected models were used to finds the hits against the EZH2. Screening was performed using the NCI library, comprising approximately 260,000 molecules. Compounds predicted active from best models were selected. The following filters have been used for selecting the hits: Filter 1—Commonly predicted compounds from both the models, Filter 2—Compounds with high probability score, and Filter 3—Compounds under the AD of high activity compounds. These compounds were further processed for molecular docking simulations. Finally, molecules with the best affinity and conformer within the active site were selected and analyzed. Three-dimensional structure of EZH2 was retrieved from Protein Data Bank (PDB ID: 5WG6). The structure was energy minimized using Discovery Studio (DS) (www.accelrys.com (accessed on 3 April 2021) Accelrys Inc., San Diego, CA, USA) to remove the steric clashes. CDOCKER program from Discovery Studio package was used for molecular docking. The binding site was defined as a sphere of 10.89 Å by using the Define and Edit Binding Site tool of DS with X, Y and Z co-ordinates of −83.47, 3.58 and −55.31, respectively.

## 5. Conclusions

Over the last decades efforts have been made to identify the potential small molecules for PRC2 via inhibiting EZH2. Emerging clinical data have provided early enough evidence for single agent activity with an acceptable safety profile for EZH2 inhibitors. We have developed machine learning-based predictive models for EZH2-inhibitor binding mechanism and to rank the activities of the molecules. Binary and multiclass models were developed using the three different machine learning approaches. Validation analyses demonstrated that these models are robust and prediction from these models is reliable and capable of predicting the true active compounds with high rank and probability score. To the best of our knowledge, this is the first report on the development of binary and multiclass predictive models for EZH2. We highlighted the potential of the classification and rigorously validated models, and the methodology was used to accurately predict and rank the activities of drugs against cancer epigenetic targets. One limitation of this study is the data size used for model building that is relatively small. Nevertheless, we believe that in future, when more EZH2 compounds’ activity data are available, these models can be used as guidance for further development of the more precise models. The validated models were used to screen the NCI library to identify the potential hits. Compounds that passed all the filters were selected for binding analyses. Finally, based on screening and molecular docking simulation, three hits with better binding affinity and interactions with EZH2 catalytic residues were selected. The purposed hits present new chemical scaffolds and could be promising starting points for the development of new optimized agents against PRC2.

## Figures and Tables

**Table 1:** List of selected descriptors used in model building.

Descriptor	Number	Name
Autocorrelation	6	AATS7v, AATS7i, AATS7s, ATSC3v, ATSC8e, GATS6s
Burden modified eigenvalues	4	SpMin3_Bhm, SpMax1_Bhv, SpMin2_Bhv, SpMax2_Bhs
Atom type electrotopological state	2	maxHBa, maxHBint5
Molecular distance edge	1	MDEC-33
Rotatable bonds count	1	RotBFrac
Topological charge	1	JGI7
Physicochemical	1	AMR
MACCSFP105	1	A$A($A)$A
MACCSFP114	1	CH3CH2A

**Table 2:** Evaluation metrics for binary models.

Descriptors Set	Methods	Accuracy	Precision	Recall	F1	AUC
ALL Descriptors	SVM	0.82	0.85	0.84	0.85	0.84
	XGB	0.78	0.75	0.70	0.78	0.88
	RF	0.79	0.77	0.78	0.79	0.89
Selected Descriptors	SVM	0.77	0.76	0.75	0.76	0.82
	XGB	0.82	0.77	0.79	0.81	0.89
	RF	0.80	0.82	0.80	0.81	0.90

SVM: Support Vector Machine; XGB: XGBoostRF; Random Forest.

**Table 3:** Evaluation metrics for multiclass models.

Descriptors Set	Methods	Metrices				AUC		
		Accuracy	Precision (Macro)	Recall (Macro)	FI (Macro)	High	Moderate	Low
ALL Descriptors	SVM	0.70	0.44	0.60	0.60	0.86	0.65	0.90
	RF	0.72	0.63	0.64	0.64	0.91	0.71	0.90
	XGB	0.73	0.65	0.67	0.64	0.87	0.73	0.92
Selected Descriptors	SVM	0.68	0.41	0.56	0.56	0.82	0.56	0.87
	RF	0.73	0.63	0.62	0.62	0.91	0.72	0.94
	XGB	0.75	0.67	0.67	0.67	0.91	0.75	0.94

SVM: Support Vector Machine; XGB: XGBoostRF; Random Forest.

**Table 4:** List of selected hits from NCI library.

Compound ID	Classifier Probability		CDOCKER Interaction Energy (Kcal/mol)
	Random Forest	XGBoost	
GSK-126	-	-	−46.55
NCI694864	0.634	0.947	−53.68
NCI670557	0.661	0.784	−47.70
NCI706726	0.622	0.905	−47.34

## Data Availability

Data is contained within the article.
